# TXNIP Links Innate Host Defense Mechanisms to Oxidative Stress and Inflammation in Retinal Muller Glia under Chronic Hyperglycemia: Implications for Diabetic Retinopathy

**DOI:** 10.1155/2012/438238

**Published:** 2012-03-18

**Authors:** Takhellambam S. Devi, Icksoo Lee, Maik Hüttemann, Ashok Kumar, Kwaku D. Nantwi, Lalit P. Singh

**Affiliations:** ^1^Department of Anatomy and Cell Biology, Wayne State University, Detroit, MI 48201, USA; ^2^Center for Molecular Medicine and Genetics, Wayne State University, Detroit, MI 48201, USA; ^3^Department of Ophthalmology, Wayne State University, Detroit, MI 48201, USA

## Abstract

Thioredoxin Interacting Protein (TXNIP) mediates retinal inflammation, gliosis, and apoptosis in experimental diabetes. Here, we investigate the temporal response of Muller glia to high glucose (HG) and TXNIP expression using a rat Muller cell line (rMC1) in culture. We examined if HG-induced TXNIP expression evokes host defense mechanisms in rMC1 in response to metabolic abnormalities. HG causes sustained up-regulation of TXNIP (2 h to 5 days), ROS generation, ATP depletion, ER stress, and inflammation. Various cellular defense mechanisms are activated by HG: (i) NLRP3 inflammasome, (ii) ER stress response (sXBP1), (iii) hypoxic-like HIF-1**α** induction, (iv) autophagy/mitophagy, and (v) apoptosis. We also found *in vivo* that streptozocin-induced diabetic rats have higher retinal TXNIP and innate immune response gene expression than normal rats. Knock down of TXNIP by intravitreal siRNA reduces inflammation (IL-1**β**) and gliosis (GFAP) in the diabetic retina. TXNIP ablation *in vitro* prevents ROS generation, restores ATP level and autophagic LC3B induction in rMC1. Thus, our results show that HG sustains TXNIP up-regulation in Muller glia and evokes a program of cellular defense/survival mechanisms that ultimately lead to oxidative stress, ER stress/inflammation, autophagy and apoptosis. TXNIP is a potential target to ameliorate blinding ocular complications of diabetic retinopathy.

## 1. Introduction

Diabetic retinopathy (DR) is the most common cause of blindness among the working age group people in the US and around the world. DR has long been considered as a microvascular disease associated with vessel basement membrane thickening, blood retinal barrier breakdown, capillary cell death, acellular capillary, neovascularization, and retinal detachment [1]. However, recent studies have demonstrated that DR is a neurovascular disease that affects both the blood vessel and neuroglia [2, 3]. Chronic hyperglycemia-associated oxidative stress and low-grade inflammation are considered to play critical roles in disease initiation and progression of diabetic complications including DR [4–6]. Yet, the molecular mechanisms underlying hyperglycemic injury and DR pathogenesis are poorly understood. Various glucose metabolic defects and abnormal biochemical pathways are activated in diabetes and under chronic hyperglycemia [7]. Recently, we demonstrated that thioredoxin interacting/inhibiting protein (TXNIP) is significantly increased both in the diabetic rat retina *in vivo* and *in vitro* in retinal endothelial cells in culture and causes pro-inflammatory gene expression for Cox-2, VEGF-A, ICAM1, RAGE, and sclerotic fibronectin (FN) [8–10]. Knockdown of TXNIP by siRNA in the retina prevents early abnormalities of DR in streptozocin-induced diabetic rats, which include inflammation, fibrosis, gliosis and apoptosis [9, 10]. 

TXNIP is an early response gene highly induced by diabetes and hyperglycemia [8, 11, 12]. TXNIP was initially identified as one of the proteins that interacts with thioredoxin (TRX) and blocks its thiol reducing function [13, 14]. TRX is a 12-kDa protein, which scavenges ROS and maintains protein cysteine sulfhydryl groups by its redox-active disulfite/dithiol sites. Recent findings further demonstrate a potential role for TXNIP in innate immunity via the NOD-like receptor-NLRP3/caspase-1 inflammasome activation and release of IL-1*β* in diabetes and oxidative stress [15, 16]. TXNIP interacts with NLRP3 leading to a multiprotein NLRP3 complex assembly and autoactivation of caspase-1. Activated caspase-1 in turn processes pro-IL-1*β* to its mature and active form leading to other pro-inflammatory gene induction and inflammation [17, 18]. Innate immune receptors TLR4 and RAGE and their endogenous ligands such as high mobility group binding protein HMGB1 and S100 calgranulins are reported to be upregulated in diabetes [19, 20]. Currently, NF-*κ*B is the only pro-inflammatory transcription factor that is implicated in retinal inflammation in diabetes [3, 4, 8, 9] while the role of other factors is undetermined. Innate immune receptors signal through activation of NF-*κ*B and AP1 transcription factors to induce the expression of various pro-inflammatory cytokines and chemokines [4–6]. 

We recently showed that TXNIP mediates IL-1*β* expression in primary Schwann cell *in vitro* as well as *in vivo* in partial sciatic nerve injury [10] by the p38 MAPK/NF-*κ*B pathway and transcription factor CREB. Similarly, TXNIP was also shown to be responsible for pro-IL-*β* expression in adipocytes by high glucose [21] and HG-mediated NLRP3 inflammasome and casapse-1 activation leads to IL-1*β* maturation [15, 16, 21]. In addition, chronic hyperglycemia may also evoke other innate host defense/survival mechanisms such as ER stress/unfolded protein response (UPR) and hypoxic responses for cell viability in an inflamed and stressful environment [22, 23]. In this regard, chronic hyperglycemia-associated TXNIP expression could potentially lead to thiol oxidation and misfolded protein accumulation in ER lumen and cause ER stress in diabetes. UPR is a highly regulated intracellular signaling pathway that prevents aggregation of misfolded proteins. ER stress is known to be mediated by three UPR branches, namely, the IRE1-XBP1, PERK-eIF-1*α*, and ATF6 pathways [22, 23]. These UPR signals provide an adaptive mechanism for proper protein folding and processing in the ER and establishment of tissue homeostasis and cell survival. However, when protein misfolding is not resolved, the ER stress and UPR trigger an apoptotic signal to remove demised cells by phagocytes. Therefore, ER stress may play a critical role in deciphering cell survival and premature demise in chronic disease and injury including DR. 

Currently, it is recognized that molecular abnormalities of DR begin early before clinically detectable pathologies appear in the retina [2–4, 24]. The pathologies once set in motion are not reversed even after glucose normalization [25, 26]. Therefore, the detection of molecular abnormalities and cellular processes that ultimately lead to later pathologies of DR is of utmost importance in developing therapeutic strategies to prevent blinding ocular complications of diabetes. Retinal Muller glia, blood vessels, and neuronal (ganglion) cells interact closely to maintain retinal tissue homeostasis and cell survival [27]. Muller cells are the primary glial type in the retina and along with microglia and astrocytes are considered as resident innate immune cells. Under stress, they become activated and produce pro-inflammatory cytokines and growth factors to restore tissue homeostasis [6]. However, in chronic diseases such as DR, retinal gliosis is prolonged, causing sustained inflammation, cell injury/death, and worsening disease. Nonetheless, a role for TXNIP in Muller glia reactivity, innate immune response, ER stress, and inflammation under chronic hyperglycemia and diabetes has not been investigated before. In this study, first, we demonstrate that TXNIP is significantly induced in the diabetic rat retina *in vivo* and mediates pro-inflammatory IL-1*β* expression and the induction of radial glial fibrillary acidic protein (GFAP) indicating Muller cell activation. Secondly, we show that chronic hyperglycemia sustains TXNIP up-regulation in Muller glia in *in vitro* culture and orchestrates a temporal program of innate host defense mechanisms that lead to cellular oxidative stress, ER stress, inflammation, and autophagy/apoptosis. These findings provide important insights as to how retinal Muller glia might respond to chronic hyperglycemia in diabetes and that TXNIP may play a critical role in glial dysfunction and disease progression in DR.

## 2. Materials and Methods

### 2.1. Materials

Tissue culture media, serum and antibiotics were purchased from Invitrogen (Carlsbad, CA). Antibodies for TXNIP and beclin 1 were obtained from MBL (Woburn, MA). Anti-S-Nitroso-Cysteine (SNO-Cys) antibody was from Sigma-Aldrich (St. Louis, MO). The LC3B antibody kit for autophagy (Cat number L10382), MitoTracker Red CMXROS (M7512), ROS detection reagent CM-H2DCFDA (C6827), and ATP assay kits were from Molecular Probes (Invitrogen). Antibodies for pro-IL-1*β*, pro-caspase-1, NLRP3 (cryopyrin), and HMGB1 are from Santa Cruz Biotechnology (Santa Cruz, CA), and VDUP1 (TXNIP), tubulin, and Actin were from Abcam (Cambridge, MA). Anti-p65 NF-*κ*B and phospho-p65 NF-*κ*B antibodies were from Millipore (Invitrogen). Electrophoresis Mobility Shift Assay (EMSA) kit for Transcription factors XBP1 and HIF-1*α* to consensus DNA was custom-made by Signosis Inc. (Sunnyvale, CA). Fluorescent-labeled secondary antibodies anti-rabbit and anti-mouse were obtained from Molecular Probes while those of the anti-goat antibodies were purchased from Abcam (Cambridge, MA). Active caspase-1 (Green FLICA Caspase-1) and Caspase-3 (Green FLICA caspase-3 & 7) were purchased from Immunochemistry Technologies (Bloomington, MN). Predesigned TXNIP siRNAs were purchased from Qiagen (SABiociences). TRIZOL for RNA isolation was from Invitrogen and PCR primers were also synthesized by Invitrogen or Applied Biosystems. First strand cDNA synthesis kit and SYBR green reagents were purchased from Biorad as well as from Applied Biosystems. Primer sequences are available upon request. 

### 2.2. Diabetes Induction of Rats

 Diabetes of adult male Sprague-Dawley rats (~275 g) was induced by intraperitoneal injection of a single dose of streptozotocin, (STZ, 65 mg/kg body weight, Sigma) dissolved in 0.01 M citrate buffer, pH 4.5 as described recently [8, 9]. The rats were treated in accordance with the principles of NIH guidelines for the Care and Use of Laboratory Animals and approved by the Institutional Animal Care and Use Committee. Intravitreal injection of siRNA targeted to rat TXNIP promoter (transcriptional gene silencing, RNAi TGS) has been described before [9] and performed in anaesthetized rats with 40 mg/kg body weight (pentobarbital) on the right eyes of diabetic rats (Treatment) and a similar volume of scramble-RNA on the left eye (Control). The injections were performed twice at day 23 and 27 and they were sacrificed at day 30. An overdose of pentobarbital (200 mg/kg weight) was given to euthanize the rats. The retina were removed, processed for immunohistological analysis or frozen immediately in liquid N_2_, and stored at –80°C until used.

### 2.3. Cell Culture

 We used a well-established rat retinal Muller cell line (rMC1) [28]. rMC1 cells were cultured in medium containing DMEM (low glucose, 5.5 mM) and Ham's F-12 (3 : 1 ratio) supplemented with 5.0% fetal calf serum (FCS) and 0.5 mg/mL gentamicin at 37°C in a humidified chamber with a 5% CO2–95% air mixture [9, 29]. Cells at 70–80% confluence were replaced with low serum overnight (0.2% serum medium). For the time course, HG (high glucose, 25 mM) with 24 h treatment was added first; then on the second day HG was added at 4, 2 h, and 0 (no addition) to respective cultures before harvesting. Thus, all cells maintain a similar condition and length of serum-deprivation during the entire experimental procedure. Similarly, for long-term cultures also, HG was added first to the 5-day culture, then after two days for 3 days, 1 day, and 0 before harvesting. Media were changed every 48 h. Cells were harvested by scrapping, snap frozen and stored at −80°C until used.

### 2.4. SDS-PAGE and Western Blotting

Proteins were extracted in RIPA buffer containing protease inhibitors and their concentrations were determined using a Coomassie Plus (Bradford) Assay Reagent from Pierce (Product number 23238) with BSA as the standard. Absorbance of blue dye of the Coomassie-protein complex was measured at 595 nm using a Gemini Microplate reader (Molecular Devices, Sunnyvale, CA). Thirty micrograms of protein extracts was subjected to sodium dodecyl sulfate polyacrylamide gel electrophoresis (SDA-PAGE) and Western blot analysis of proteins was performed as described previously [30]. Santa Cruz antibodies were used at 1 : 200 dilutions while antibodies from other sources were diluted at 1 : 1000 dilutions. Secondary antibodies were used at 1 : 3000 dilutions. ECL used to detect the immunoreactive bands.

### 2.5. Real-Time Quantitative PCR

 Total RNAs were isolated by TRIZOL method and first-strand cDNAs (from 1 *μ*g RNA) were synthesized in 20 *μ*L volume using the Bio-Rad iScript cDNA synthesis kit [8, 12]. Messenger RNA expression was analyzed by real-time quantitative PCR using the Bio-Rad Chromo 4 detection system and SYBR Green PCR Master Mix from Bio-Rad or Applied Biosystems (Foster City, CA). Primers were designed using Primer Express v 2.0 (Applied Biosystems) and synthesized by Invitrogen (Carlsbad, CA). Primers for the real-time Q-PCR will be available upon request.

The real-time PCR reaction mixture contained 1X SYBR Green PCR Master Mix, 400 nM forward and reverse primers, and 2 *μ*L cDNA in a final volume of 25 *μ*L. The PCR cycling was programmed as 95°C for 15 s, 55°C for 30 s and 72°C for 30 s 40 cycles followed by the construction of a melting curve through increasing the temperature from 60°C to 95°C at a ramp rate of 2% for 20 min. The real-time PCR samples were evaluated using a single predominant peak as a quality control. Ct values were used to calculate the relative expression level of mRNAs that were normalized to actin [8, 12].

### 2.6. Determination of Cell Viability

We used MTT assay to measure cell viability at different time periods after HG addition. MTT assay for cell viability was performed in 48 well plates and with 0.5 mg/mL MTT in each well as previously described in our laboratory [31, 32]. rMC1 cells (1 × 10^4^ cells) were grown to 70% confluence and serum-starved overnight and treated with HG. MTT was added for 3 h at the end of the experiment, the media was removed, and cells were kept in 100 *μ*L of DMSO for 10 minutes. The resulting color was diluted with 500 *μ*L of distilled H_2_O and detected at 570 nm using a Gemini Microplate reader (Molecular Devices, Sunnyvale, CA). 

### 2.7. Intracellular Reactive Oxygen Species (ROS) Measurement

The formation of intracellular ROS in rMC1 cells was detected as described before [8, 12] by using the fluorescent probe, 5-(and-6)-chloromethyl-2′,7′-dichlorodihydrofluorescein diacetate, acetyl ester (CM-H_2_DCFDA). This dye can enter living cells by passive diffusion it is nonfluorescent until the acetate group is cleaved off by intracellular esterase, and oxidation occurs within the cell. Approximately 1 × 10^5^ cells/mL were cultured in 24 well plates, serum-starved overnight, and glucose was added for the specified time period. Then, CM-H_2_DCFDA (10 *μ*M) was incubated for 60 min at 37°C. The medium with the dye is aspirated (to remove the extracellular dye), washed with PBS (3x), and then the PBS is added to cells. The fluorescence was measured in a Gemini Fluorescent Microplate Reader (Molecular Devices) with the bottom read scanning mode at 480 nm excitation and emission at 530 nm. 

### 2.8. Immunohistochemistry (IHC)

**(i) Retinal Tissue**

Immunohistology of retinal sections was similar to those described recently [9]. The retinas were fixed in the eyecups with 4% paraformaldehyde in 0.1 M phosphate buffer (PB) for 20 min. The retinas were cryoprotected in a sucrose gradient (10%, 20%, and 30% w/v in PB, resp.). Cryostat sections were cut at 16 *μ*m in Tissue Tek (OCT mounting medium). Retinal sections were blocked for 1 h in a PB solution that contained 5% Chemiblocker (Chemicon, Temecula, CA), 0.5% Triton X-100, and 0.05% sodium azide. The primary antibodies were diluted in the same solution (1 : 100 to 1 : 200 dilution depending on the antibody) and applied overnight at 4°C followed by appropriate secondary antibodies (1 : 600 dilutions) conjugated with Alexa Fluor 488 or Alexa Fluor 594 for 2 hours at room temperature. After washing with PB again, rMC1 cells were mounted with an aqueous mounting medium with anti-fade agent with DAPI and sealed with nail polish. The images were captured by an OLYMPUS BX 51 fluorescence microscope, which is fitted with a triple DAPI/FITC/TRITC cube, a DP70 digital camera, and image acquisition software. Some images were also captured with a Zeiss Apotome microscope with Z-section (Zeiss, Oberkochen, Germany). Similar magnification (400x) and exposure time were maintained throughout for comparing images unless otherwise mentioned.

**(ii) rMC1 Cells**

Cells were grown either in four-chambered tissue culture glass slides (NUNC, Naperville, IL) and exposed to HG for 5 days as described [32]. Cells were fixed with freshly prepared paraformaldehyde (4%) for 2 h in ice or 4°C overnight, washed 10 min each with PB (3 times), and blocked with 5% horse serum in PB for 1 h at room temperature. Following a 30-minute wash with PBS, cells were incubated with primary antibodies (1 : 100 dilutions) overnight at 4°C in a humidified chamber. After washing with PB, cells were further incubated with corresponding secondary antibodies conjugated with Alexa Fluor 488 or Alexa 594 at 1 : 500 dilutions for 1 h at 37°C in a darkened humidified chamber. The cell-associated fluorescence was observed in Olympus BX51 fluorescence microscope.

### 2.9. Cytochrome *c* Oxidase and ATP Assays

 The mitochondrial oxidative phosphorylation (OxPhos) process consists of the electron transport chain (ETC) and ATP synthase, and it provides more than 90% of cellular energy. The terminal enzyme of ETC is cytochrome *c* oxidase (CcO). CcO transfers electrons from cytochrome *c* to oxygen, which is reduced to water. In intact mammalian cells, this reaction is the proposed rate-limiting step of the ETC under physiological conditions [33]. Therefore, CcO is an ideal marker enzyme of OxPhos and was analysed in this study.

**(i) CcO Activity**

The oxygen consuming capacity of CcO- analyzed in a closed chamber equipped with a micro-Clark-type oxygen electrode (Oxygraph system, Hansatech, Norfolk, England) as previously described [34, 35]. Briefly, frozen cells were solubilized in 10 mM K-HEPES (pH 7.4), 40 mM KCl, 1% Tween 20, 1 *μ*M oligomycin, 1 mM PMSF, 10 mM KF, 2 mM EGTA, and 1 mM Na vanadate. CcO activity was measured in the presence of 20 mM ascorbate and by addition of increasing amounts of cow heart cytochrome *c*. Oxygen consumption was recorded on a computer and analyzed with the Oxygraph software. Protein concentration was determined with the DC protein assay kit (Bio-rad). CcO-specific activity is defined as consumed O_2_  (*μ*M)/min/mg total protein.

**(ii) ATP Assay**

Determination of ATP levels using the bioluminescent method was performed as described previously [34, 35]. ATP was released from rMC1 cells maintained in 24 well plates using the boiling method. Two hundred fifty *μ*l TE buffer (50 mM Tris-Cl, pH 7.4, 4 mM EDTA) was added to the wells and scrapped off. Cells were immediately transferred to a boiling water bath for 4 min. Samples were put on ice and sonicated briefly and centrifuged. ATP concentration was determined in the supernatant with an ATP bioluminescence assay kit (Invitrogen) according to the manufacturer's manual. Relative fluorescence units (RLUs) were detected in a Luminometer (Promega, Madison, WI). Experiments were performed in triplicates for each condition and data were standardized to the protein concentration using the Biorad protein assay kit.

### 2.10. Gel-Shift Assay

The gel-shift or electrophoretic mobility-shift assay (EMSA) provides a simple and rapid method for detecting DNA-binding activity of proteins. We investigated the sXBP1 and HIF-1*α* activity of rMC1 nuclear proteins by using a commercially available gel-shift assay kit from Signosis (Sunnyvale, CA) according to manufacturer's instructions and as described before [32]. After incubation at 16°C for 30 min with biotin-labeled probes and nuclear extracts (5.0 *μ*g protein) in a PCR machine, the protein-DNA complexes were subjected to 6.5% nondenaturing polyacrylamide gels prepared with TBE gel formulation [32]. The gels were run at 100 V for 1 h until the Bromophenol blue dye front is three quarters down the gel, using 0.5x TBE running buffer. For competitive assays, excess cold probes were added to the reaction. Streptavin-HRP and ECL were used to detect and capture the reactive bands using a Cell Bioscience FluorChem E System (Santa Clara, CA).

### 2.11. Statistical Analysis

Results are expressed as means +/−  SE. Student's *t*-test or one-way ANOVA followed by Bonferroni Post Hoc Test was used to compare differences between treatment conditions [8, 9]. A preset *P* value of <0.05 was considered statistically significant.

## 3. Results

### 3.1. Diabetes Induces TXNIP Expression, Muller Glia Reactivity, and Proinflammatory Gene Expression in the Rat Retina

**Figure 1:**
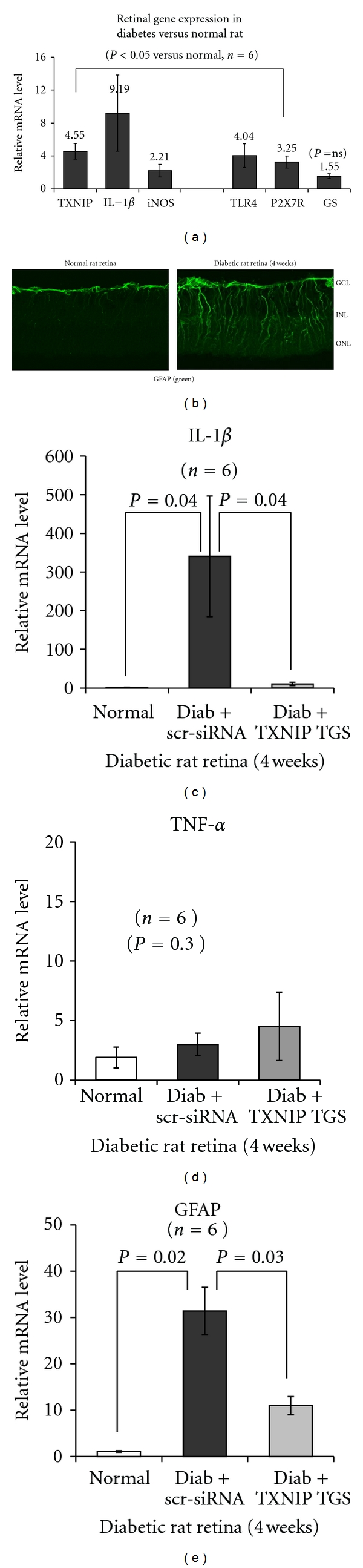
Diabetes induces TXNIP and pro-inflammatory gene expression and glia reactivity in the rat retina. (a) Messenger mRNA levels for TXNIP (4.55-fold ± 0.96) and pro-inflammatory IL-1*β* (*9.19* ± *4.62*), iNOS (2.22 ± 0.76), and pattern recognition receptors TLR4 (4.04 ± 1.43) and P2X7R (3.26 ± 0.73) are increased significantly (*P* < 0.05, *n* = 6) in the retina of diabetic rats (4 weeks) when compared with the normal retina. GS is marginally increased (1.55 ± 0.29) but not significant (*P* = 0.3). (b) GFAP staining, a marker of gliosis, is also increased radially throughout the neuroretina in the diabetic rat versus the normal retina, suggesting Muller glia activation. GCL: ganglion cell layer; INL: inner nuclear layer; and ONL: outer nuclear layer. (c–e) TXNIP knockdown by siRNA targeted to the promoter (RNAi TGS, [9]) reduces IL-1*β* and GFAP mRNA levels in the diabetic retina as compared to the scr-siRNA-treated diabetic rat retina. Under these conditions (4 weeks of diabetes induction in rats), we did not see an increase in retinal TNF-*α* mRNA level.

 We have recently shown that TXNIP expression is increased in the retina of STZ-induced diabetic rats at both 4 and 8 weeks of diabetes duration [8, 9] and mediates inflammation, gliosis/fibrosis, and apoptosis of retinal cells. In this study, we further support our previous findings by showing that the expressions of TXNIP, pro-inflammatory IL-1*β*, iNOS, and pattern recognition receptors TLR4 and P2X7R in the retina are elevated at 4 weeks of diabetes (Figure 1(a)). On IHC, we observed that the increased pro-inflammatory gene expression is associated with enhanced GFAP staining, a marker for glia activation, in the entire length of the neural retina from diabetic rats when compared with the nondiabetic normal rat retina (Figure 1(b)). Specifically, in the normal rat retina, GFAP staining is restricted to the inner limiting membrane of Muller glia end-feet and ganglion cell layer (GCL), however, in the diabetic rat retina, GFAP filaments extend from GCL to inner nuclear (INL), outer nuclear layer (ONL), and the inner and outer plexiform layers suggesting retinal injury and inflammation. Knockdown of TXNIP by intravitreal injection of promoter-targeted siRNA (RNAi TGS) further reduces IL-1*β* and GFAP up-regulation significantly (*P* < 0.05, *n* = 6) in the diabetic rat retina (Figures 1(c) and 1(e)). Under these conditions, we did not observe an enhancement of TNF-*α*,  another pro-inflammatory cytokine, in the diabetic retina (Figure 1(d)). TNF-*α* has previously been shown to induce at early at 1-2 weeks of STZ-induced diabetic rat retinas and then suppresses until late stages of diabetes duration where it may play a critical role in retinal inflammation and BRB breakdown [26]. Thus, the results demonstrate that there is an early inflammatory response and gliosis in the diabetic retina. Retinal Muller glia forms a close association with neuron and blood vessel and plays a critical function in maintaining retinal homeostasis in health and disease. Therefore, we propose that Muller glia reacts to retinal injury both in retinal vessels and neurons in diabetes and produces pro-inflammatory and survival factors to restore tissue homeostasis. 

### 3.2. HG Induces TXNIP Expression and Innate Immune Responses in rMC1 in Culture

**Figure 2:**
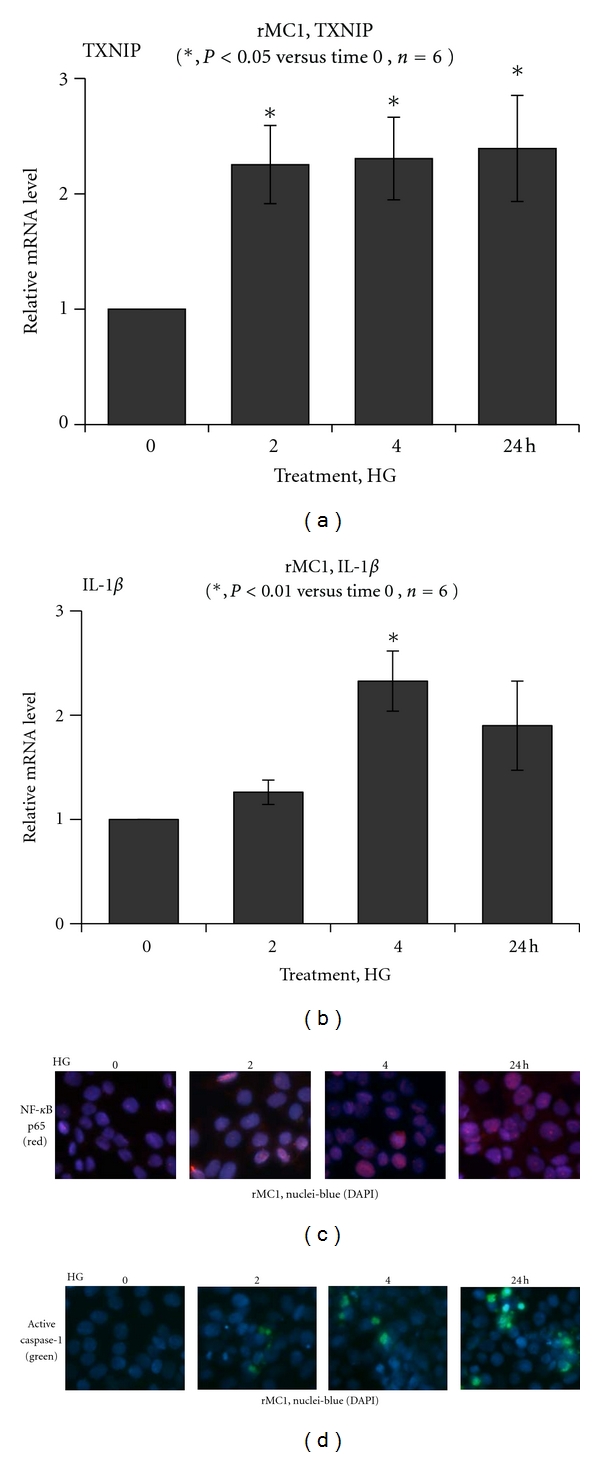
HG induces TXNIP expression and evokes the host innate immune response in rat Muller glia. Quantitative RT-PCR for (a) TXNIP and (b) IL-1*β* mRNA expression in rMC1 is shown. HG increases TXNIP mRNA at 2–24 h (*P* < 0.05 versus time 0, *n* = 6) while pro-IL-1*β* mRNA is increased at 2 and 4 h (*P* < 0.05 versus time 0, *n* = 6) and then reduces at 24 h. (c) IHC detects a time-dependent accumulation of the p65 subunit of NF-*κ*B in the nucleus in rMC1 under HG. (d) Active caspase-1 staining is increased in rMC1 using the caspase-1 FLICA probe. A representative of *n* = 3 is shown here.

 We have shown previously that TXNIP expression and Muller glia activation occur early in the diabetic retina [9]. So far, a role for TXNIP in Muller cell activation and pro-inflammatory gene induction under HG exposure has not been investigated. Therefore, we undertook a series of experiments to examine the temporal response of rMC1 to chronic hyperglycemia in culture. We show that HG exposure in rMC1 induces a significant (*P* < 0.05, *n* = 6) increase in TXNIP mRNA expression (2–24 h) when compared with low glucose (5.5 mM, LG) indicated as time 0 control (Figure 2(a)). We have previously shown that TXNIP mediates pro-IL-1*β* expression in primary Schwann Cells via the NF-*κ*B-dependent pathway [10]. Therefore, we examined if TXNIP expression correlates with pro-IL-1*β* induction in rMC1 under HG. As shown in Figure 2(b), HG increases IL-1*β* mRNA expression at 2 and 4 h (*P* < 0.01) and then reduces at 24 h to the level of the control at 0 time. Furthermore, nuclear NF-*κ*B level is also enhanced by HG in rMC1 from 2 to 24 h (Figure 2(c)), which correlates with activation of caspase-1 in rMC1 (Figure 2(d)). Active caspase 1 is responsible for processing pro-IL-1*β* (34-kDa) to an active mature form of IL-1*β* (17-kDa).

**Figure 3:**
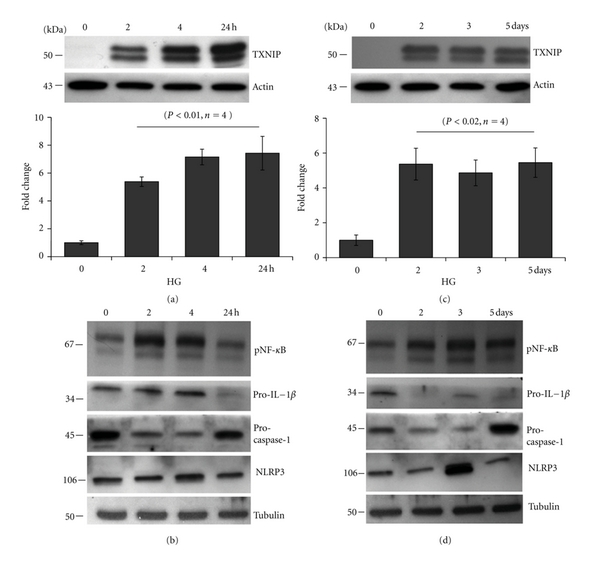
Chronic hyperglycemia persistently upregulates TXNIP expression in rMC1 and induces IL-1*β* and NLRP3 inflammasome activation. rMC1 cells were cultured under HG for (a and b) 0–24 h or (c and d) 0–5 days and TXNIP proteins were detected by Western blotting. For this, cell extracts were prepared in RIPA buffer and 30 *μ*g protein was analyzed on 12% SDS-PAGE and Western blot for cytosolic TXNIP, Pro-IL-1*β*, NLRP3, and pro-caspase-1 and the nuclear level of phosphorylated p65 at serine residue 276 (S276) of NF-*κ*B. ECL detected the immunoreactive bands. Actin and tubulin were used as controls for protein loading. A representative blot for each protein is shown here from *n* = 3-4.

We further examine whether the protein levels of TXNIP and pro-IL-1*β* protein are induced by HG and if the levels of NLRP3 and caspase-1 are important for processing pro-IL-1*β* to its mature form in a time-dependent manner. TXNIP expression is low in rMC1 under LG while HG upregulates TXNIP (~4-folds) significantly from 2 h to 5 days (Figures 3(a) and 3(c)). Nuclear level of phosphorylated p65 subunit of NF-*κ*B increases at 2 and 4 h but reduces at 24 h (Figure 3(b)); however, it elevates again at day 2 through day 5 (Figure 3(d)). Pro-IL-1*β* is also increased at 2 and 4 h of HG exposure and reduces at 24 h, and again induces at 3 days of HG (Figures 3(c) and 3(d)), which correlates with NLRP3 expression. Procaspase 1 level is lower at 2 and 4 h and upregulated at 24 h (Figure 3(c)). This is in agreement with the increased level of active caspase-1 at 24 h shown in Figure 2(d). Pro-caspase-1 level rises again at 5 days of HG exposure (Figure 3(d)). The results demonstrate that TXNIP is a glucose-sensitive gene and its expression remains upregulated when hyperglycemia persists. On the other hand, the innate immune response to chronic hyperglycemia by IL-1*β*, NLRP3 and caspase-1 inflammasome is cyclical.

### 3.3. Chronic Hyperglycemia Induces ROS Generation and Reduces Cellular ATP in rMC1

**Figure 4:**
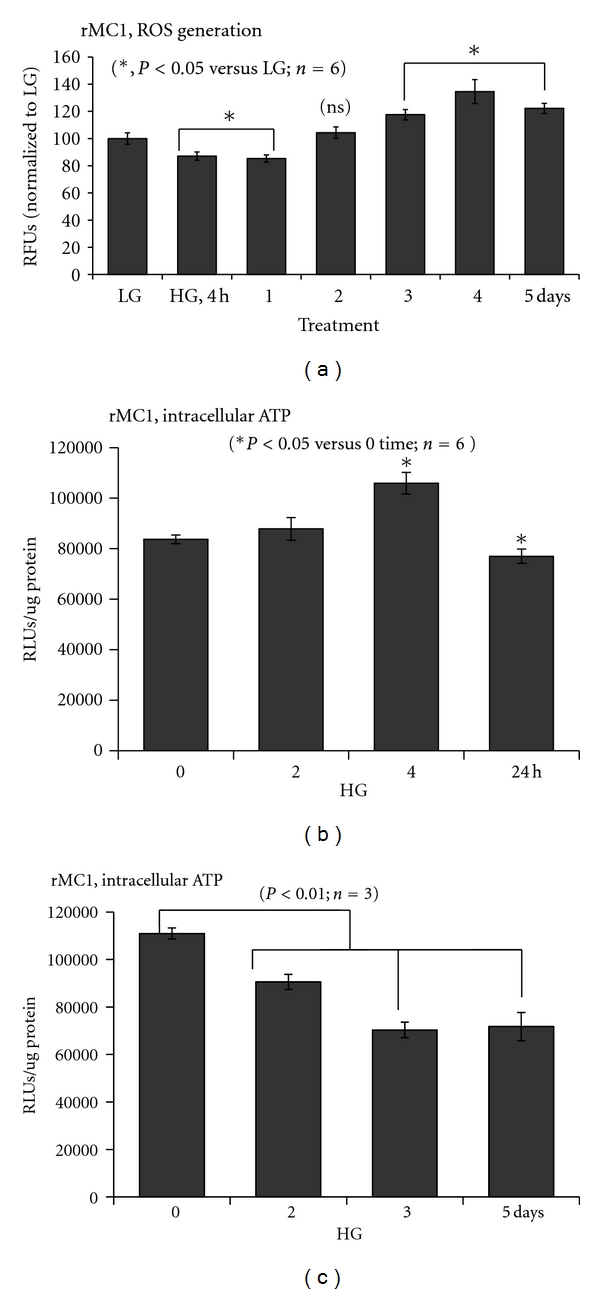
Time-dependent effect of HG on ROS and ATP generation in rMC1. (a) Reactive oxygen species (ROS) generation was measured using a fluorescence probe, CM-H2DFA. ROS level is reduced at both 4 and 24 h significantly (*P* < 0.05, *n* = 6) but rises at day 3 to 5 (*P* < 0.05, *n* = 6). (b-c) ATP level was determined in rMC1 cells by a Renilla-based luminescence assay kit (Invitrogen). Intracellular ATP is increased at 4 h (*P* < 0.05, *n* = 6) and reduces at 24 h as well as at day 2 through 5 (*P* < 0.01, *n* = 3). However, at day 3 and 5, the level of ATP is further reduced to ~65% of the control.

 We have observed previously that HG sustains TXNIP and induces IL-1*β* expression. TXNIP is a pro-oxidative stress protein through its interaction/inhibition of the thiol reducing activity and ROS scavenging capacity of TRX. In addition, extracellular ATP release has been shown to involve in NLRP3 inflammasome assembly, and IL-1*β* processing. Therefore, we examined a time-dependent generation of intracellular ROS and ATP as well as extracellular ATP release in rMC1 upon HG exposure. HG significantly reduces ROS levels early at 4 and 24 h (*P* < 0.05 versus LG, *n* = 6) and then rises at day 2 though not significant (Figure 4(a)). However, at day 3 of HG exposure, rMC1 cells produce significantly higher levels of ROS (*P* < 0.05, *n* = 6) than LG and sustain up to day 5. 

Excess nutrient (glucose availability) could enhance glucose metabolic flux through glycolysis and mitochondrial OxPhos via the electron transport chain (ETC) and ATP production. Therefore, we measured temporal changes in intracellular ATP levels in rMC1 after HG exposure. As shown in Figure 4(b), the intracellular ATP concentration is significantly (*P* < 0.05, *n* = 4) enhanced at 4 h and reduces at 24 h when compared with time 0 control. The reduction in ATP persists at day 2 through 5 (*P* < 0.01 versus time 0 control, *n* = 3). At day 3 and 5 of HG, the level of ATP is further reduced to ~65% of the control ATP level at LG (Figure 4(c)). In case of the extracellular ATP level, there is a small but significant increase at 4 and 6 h and then at day 1 onwards their levels are reduced though not significant (unpublished data). These results indicate that an initial increase in ATP generation via the mitochondrial OxPhos may result in ROS generation. Initially, this may induce an antioxidant response leading to a reduction in ROS level. However, as hyperglycemia persists, ROS overwhelms the anti-oxidant capacity.

### 3.4. Mitochondrial Electron Transport Chain (ETC) Protein Cytochrome *c* Oxidase Activity (CcO) and MTT Cell Viability Are Maintained under HG

**Figure 5:**
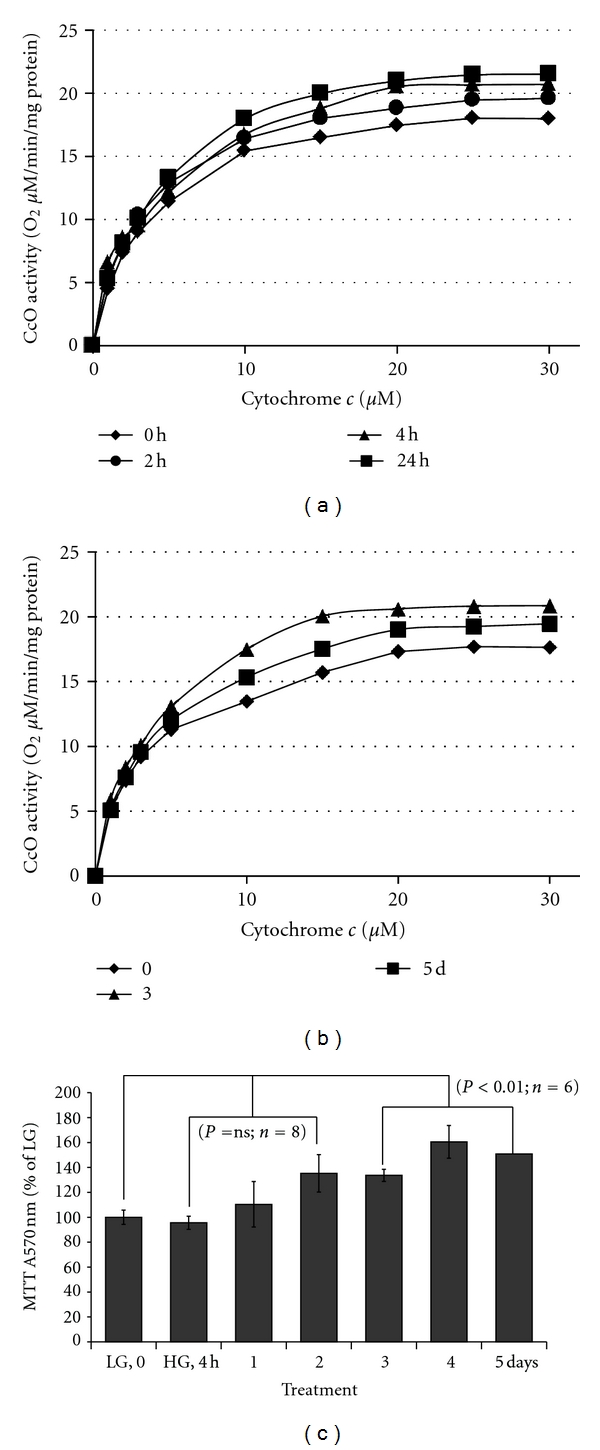
HG effects on mitochondrial electron transport chain (ETC) enzyme activity in rMC1. (a-b) Cytochrome c oxidase (CcO) activity was determined by increasing the amount of substrate cytochrome *c* in a polarographic method as described in Methods. CoO activity is defined as consumed O_2_ [*μ*M]/min/protein [mg]. Oxidative metabolism was increased after incubation in high glucose medium as was seen by an incremental increase of CcO activity at 24 h as well as at 3 and 5 day by ~12%. Shown are representative experiments (*n* = 3; standard deviation <4% at maximal turnover). (c) MTT Assay for rMC1 cell viability was measured in 48-well cultures at various time periods of hyperglycemia exposure. MTT activity was not significantly altered up to 2 days; however at day 3 to 5, there is a significant (*P* < 0.01, *n* = 6–8) increase versus LG (time 0 control).

 As described previously, ROS generation and ATP reduction occur in rMC1 cells under chronic HG. Therefore, the mitochondrial ETC function, especially the oxygen consumption capacity of CcO activity at Complex IV, may be reduced. We measure the O_2_ consuming capacity of CcO in an *in vitro* assay using a closed oxygen chamber. In contrast to our prediction, the CcO activity is unaffected by chronic HG exposure of rMC1 (Figures 5(a) and 5(b)). In addition, we did not observe an initial sigmoidal rate of the CcO activity using different protein concentrations of cytochrome c in the O_2_ consuming assay, indicating that, under these experimental conditions, CcO may not be regulated by posttranslational modification as previously described [34].

We next examined whether cell viability is reduced by ROS and ATP in rMC1 cells using MTT assay. An increase in MTT absorbance measures the reductase activity of mitochondrial succinate dehydrogenase at complex II, which is frequently used to monitor cell viability. As shown in Figure 5(c), MTT activity is not affected by HG exposure up to 2 days and increases at day 3 through 5 (*P* < 0.01, *n* = 6). These results suggest that rMC1 maintains a functional mitochondria and viability in spite of the ROS generation and ATP depletion. To further ascertain that HG maintains rMC1 viability under increased ROS, ATP release, and IL-1*β* expression, we treated rMC1 cells with exogenous H_2_O_2_ (0.2 mM), ATP (0.1 mM), and IL-1*β* (10 ng/mL) with or without HG. We observed that H_2_O_2_, ATP, and IL-1*β* increase ROS levels significantly (*P* < 0.05, *n* = 6) at 4 to 96 h and reduce cell viability as indicated by reduced MTT levels under LG at 48 h (unpublished data). However, when HG is added, the effect of H_2_O_2_, ATP, and IL-1*β* on MTT activity is nullified. This occurs even when HG increases the generation ROS further with H_2_O_2_, ATP, or IL-1*β* in combination. Therefore, these findings suggest that rMC1 activates a cellular defense and survival mechanism(s) under HG and oxidative stress, and TXNIP may play a role in this process.

### 3.5. HG Increases Autophagy/Mitophagy, Inflammation, and Apoptosis in rMC1 Cells

**Figure 6:**
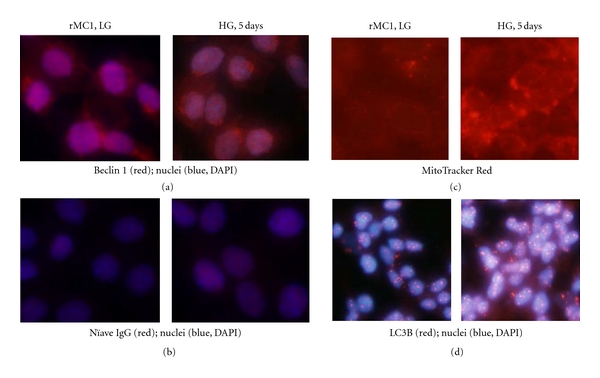
HG activates autophagy/mitophagy in rMC1. Cells were grown in HG for 5 days under low serum conditions in a four-well slide chamber with media changing for every 48 h and 24 h for the final day. Cells were fixed and stained for (a) beclin 1 antibody and (b) a naïve IgG for control antibody reaction. There are more beclin 1 puncta in HG than in LG. (c) MitoTracker Red staining also reveals more mitochondrial puncta in HG than in LG. (d) LC3B antibody staining shows an increased LC3B level and autophagic puncta formation in rMC1 under HG than in LG. The pictures are representative of 3 separate experiments.

We observed that HG increases TXNIP expression and ROS generation and sustained ATP reduction in rMC1 cells. Nonetheless, the mitochondrial CcO enzyme activity and cell viability are maintained. Therefore, we hypothesize that a cell survival program is evoked under chronic hyperglycemia and oxidative stress in rMC1. Excess ROS production causes protein misfolding/aggregation and organelle damage (e.g., mitochondria). Autophagy is a cellular survival mechanism for long-lived fully differentiated cells and for phagocytes under cellular stress to remove damage organelles and their regeneration [36, 37]. Beclin 1 is an autophagy initiating protein that interacts with prosurvival bcl2 and bcl-xL [38]. Under oxidative stress, beclin 1 is released from bcl2 and activates the autophagic process, which further activates the late phage autophagosome marker, light chain LC3B [39]. Therefore, we examined beclin 1 expression in rMC1 under chronic hyperglycemia at day 3 and 5. As shown in Figure 6(a), beclin 1 staining is marginally increased in rMC1 under HG than in LG, however there are more beclin 1 punctae in the cytosol and over the nuclei suggesting activation of an autophagic process. This beclin 1 puncta is not seen up to day 3 (not shown). 

Next, we used a mitochondrial staining dye, MitoTracker red, to examine the mitochondrial morphology. We observed an enhanced mitochondrial staining as well as the formation of larger mitotracker puncta (Figure 6(c)), indicating that autophagy/mitophagy may occur in rMC1 under chronic hyperglycemia. Subsequently, using an autophagy assay kit for LC3B staining, we show that HG increases the number and size of LC3B puncta formation (LC3BII, active form) in rMC1 when compared with LG (Figure 6(d)). Smaller LC3B punctae are also present in rMC1 after 5 days of LG under low serum medium. In addition, we observed that at 8 weeks of diabetes, formation of LG3B puncta occurs in the retina and they colocalize with TXNIP (unpublished data).

The mechanism of autophagy/mitophagy initiation under chronic hyperglycemia, TXNIP, and ROS is not fully understood at the present time. However, the thiol oxidation of the nuclear high mobility group box protein 1 (HMGB1) under oxidative stress has been implicated in its nuclear export and cytosolic interaction with beclin 1 to release from bcl2 and to initiate an autophagic process [39]. Therefore, we examined levels of TXNIP and HMGB1 in the nucleus and cytosol. We observed that TXNIP expression in the nucleus is also increased in rMC1 under HG (unpublished data) in addition to their cytosolic expression as observed in Figure 3. HMGB1 levels, both in the nucleus and in the cytosol, are not altered at 0–24 h but decrease at day 2 and 3 in the cytosol and then increase again at day 5. Furthermore, we observed that protein cysteine (thiol) nitrosylation (SNO) is enhanced in HG as measured by an anti-SNO antibody on IHC, and SNO colocalizes with HMGB1 (unpublished data). In addition, beclin 1 and HMGB1 colocalize in the cytosol. These results show that autophagy/mitophagy is activated in rMC1 under HG and that beclin 1 and oxidized HMGB1 may play a role in initiating the process.

**Figure 7:**
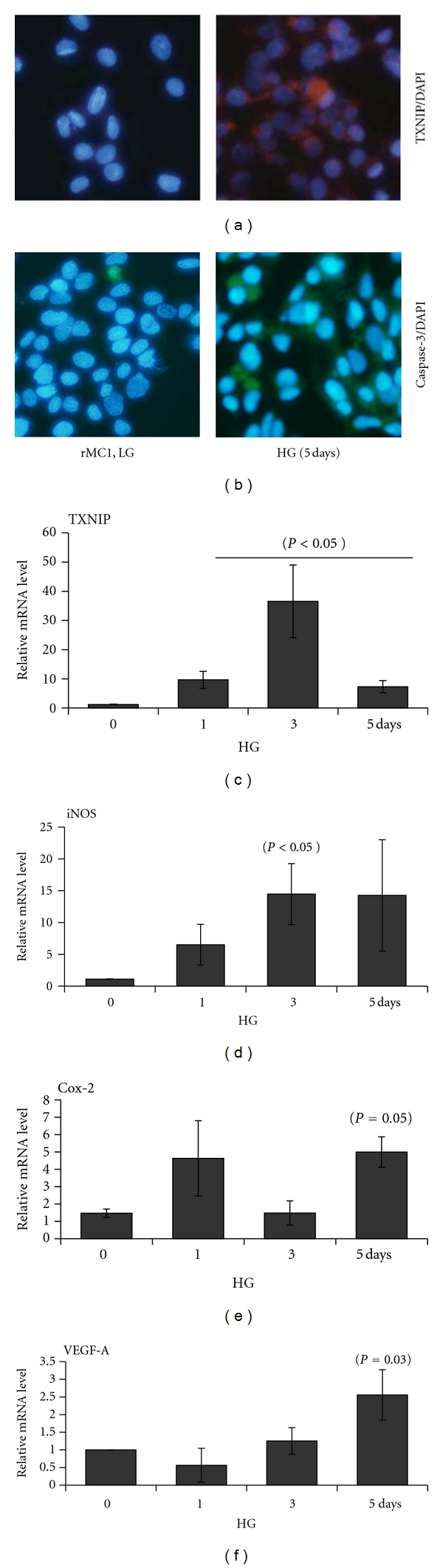
HG-induced TXNIP expression correlates with proapoptotic caspase-3 expression in rMC1. (a) IHC of TXNIP. Txnip staining is enhanced in rMC1 under HG exposure for 5 days, which correlates with increased (b) proapoptotic caspase 3 staining in similar duration of HG using a caspase-3 FLICA staining kit. A representative of *n* = 3 is shown here. (c–f) HG induces TXNIP and pro-inflammatory gene expression in rMC1. Total RNA was isolated with TRIZOL and mRNA levels for (c) TXNIP, (d) iNOS, (e) Cox-2, and (f) VEGF-A were measured by qRT-PCR at various time periods of HG exposure (*n* = 3-4). *P* values were compared against respective controls at time 0.

Autophagy/mitophagy is initially a mechanism for cell survival; however excessive and continuous autophagy may remove vital proteins and organelles and induce cell apoptosis [36, 37]. Therefore, we examined whether proapoptotic caspase-3 is activated under chronic HG in rMC1. As shown in Figures 7(a) and 7(b), HG increases TXNIP and caspase-3 staining in rMC1 when compared with LG, suggesting cells undergoing a path of apoptotic cell death. Furthermore, injured and dying cells induce the expression of pro-inflammatory genes significantly (*P* < 0.05) for TXNIP (~36.55 ± 12.45 at day 3), iNOS (14.47 ± 4.8 at day 3), Cox-2 (4.49 ± 0.88 at day 5) and VEGF-A mRNA (2.15 ± 0.27 day 5) (Figures 7(c)–7(f)). These results suggest that under HG and oxidative stress, rMC1 cells promote an inflammatory and autophagy/mitophagy response to remove defective organelles.

### 3.6. HG Evokes an Early ER Stress and a Later Hypoxic-Like Response in rMC1

**Figure 8:**
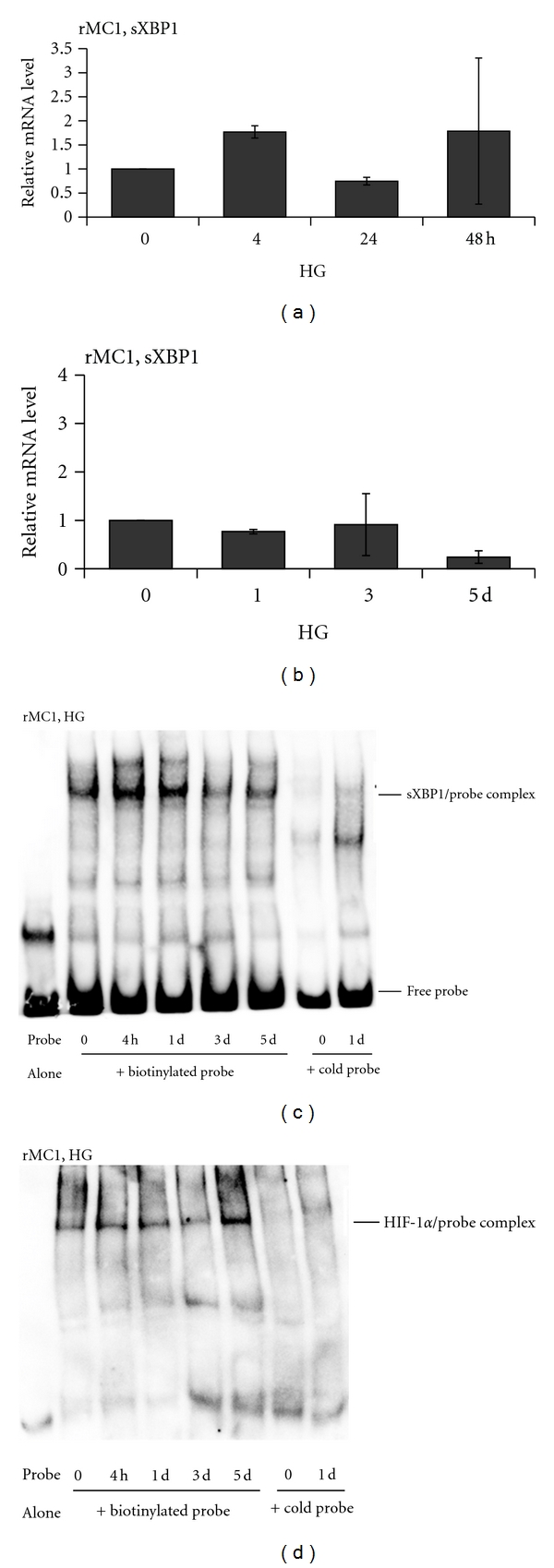
HG induces an early ER stress and a later hypoxia-like response in rMC1. Messenger RNA for the spliced form of ER stress marker XBP1 (sXBP1) was measured in rMC1 at various time periods (a) 0–24 h and (b) 0–5 days after HG exposure by qRT-PCR There is no significant change in sXBP1 mRNA (*p* = *ns*, *n* = 3) when compared with respective controls at time 0. (c) The DNA-binding activity of sXBP1 in rMC1 was further measured by EMSA using nuclear extracts (5 *μ*g) and biotin-labeled DNA probes with or without a competitive cold DNA probe as described in Methods. Protein-DNA complexes were detected by streptavin-HPR and ECL. In the presence of competitive cold probe, the sXBP1 reactive band is abolished indicating the specificity of the sXBP1-DNA binding (last two lanes). Probe alone without the nuclear extract was also run as a control (first lane). The sXBP1 activity is increased at 4 h and 1 day in rMC1 by HG and then returns to the control level observed at day 0. (d) The DNA-binding activity of the hypoxic response factor HIF-1*α* in nuclear extracts was also measured using a biotin-labeled HIF-1*α* binding consensus DNA probe. HIF-1*α* activity is not increased up to day 3 in rMC1 by HG but enhances at day 5. Competitive cold probes block the DNA-binding of HIF-1*α*, indicating the specificity of the binding assay. The images are representative of *n* = 3 in both (c) and (d).

As shown above in Figure 3, TXNIP upregulation is sustained in rMC1 cells under conditions of HG. TXNIP is a pro-oxidant and pro-inflammatory protein [8, 12]. However, we observed at 4 and 24 h after HG exposure in rMC1 that TXNIP up-regulation is associated in fact with a significant decrease in ROS level (Figures 3 and 4). Therefore, we hypothesized that the early induction of ATP generation in rMC1 by HG (Figure 4(b)) might induce a mild increase in mitochondrial ROS (and possibly an increase in protein synthesis) that could potentially cause an ER stress and unfolded protein response, such as the IRE1/XBP1 branch. Spliced form sXBP1 (an active transcription factor) of XBP1 mRNA is translated and translocated to the nucleus where it induces the expression of antioxidant genes (e.g., superoxide dismutases and catalase) [22, 23]. Indeed, expression of SOD1 and catalase effectively increases the cellular antioxidant capacity [40]. Therefore, we examined levels of sXBP1 mRNA. We did not observe a significant alteration in sXBP1 mRNA in HG when compared with LG although there is an increasing trend at 4 h (Figures 8(a) and 8(b)). However, the DNA-binding activity of the nuclear sXBP1 is increased in EMSA (Figure 8(c)) at 4 and 24 h by HG and reduces at day 3 and 5. These results suggest that there is an early ER stress response in rMC1 under HG, which may effectively scavenge ROS (Figure 4(a)). However, as the ROS continues to accumulate under sustained hyperglycemia, the anti-oxidant capacity is overwhelmed and an oxidative stress occurs at day 3 to 5 when XBP1 activity is reduced. Activation of other branches of UPR/ER stress (e.g., PERK-eIF-2 and ATF6) is yet to be investigated. Nonetheless, sustained ROS/RNS generation will cause excessive organelle damage and protein aggregation leading to apoptotic signal induction (Figure 7(b)), which may involve ER stress mediated apoptotic signals, CHOP (transcription factor CEBP homologous protein), and Bim (Bcl-2 interacting mediator of cell death) expression [41]. Further studies are being pursued to test this hypothesis. 

Under persistent hyperglycemia, ROS, and sustained ATP depletion (Figure 4(b)), a hypoxic-like response in rMC1 may evoke and induce pro-inflammatory gene expression for iNOS, VEGF-A, and Cox-2 as seen in Figures 7(d)–7(f). Therefore, we measured the activity of hypoxia inducible transcription factor (HIF) 1*α* in EMSA and the result is shown in Figure 8(d). There is no increase in DNA-binding activity of HIF-1*α* in rMC1 up to day 3 by HG. However, at day 5 the HIF-1*α* DNA-binding activity is enhanced, which correlates positively with the expression of its down stream gene target, vascular permeability, and proangiogenic VEGF-A (Figure 7(f)). These results suggest strongly that chronic HG evokes an initially time-dependent early innate immune and ER stress response in rMC1, followed by ATP reduction, ROS accumulation, and HIF-1*α* activation leading, at least in part, to pro-inflammatory and proangiogenic gene expression.

### 3.7. Knockdown of TXNIP by siRNA Blocks ATP Reduction, ROS Generation, and LC3B Activation by HG in rMC1

**Figure 9:**
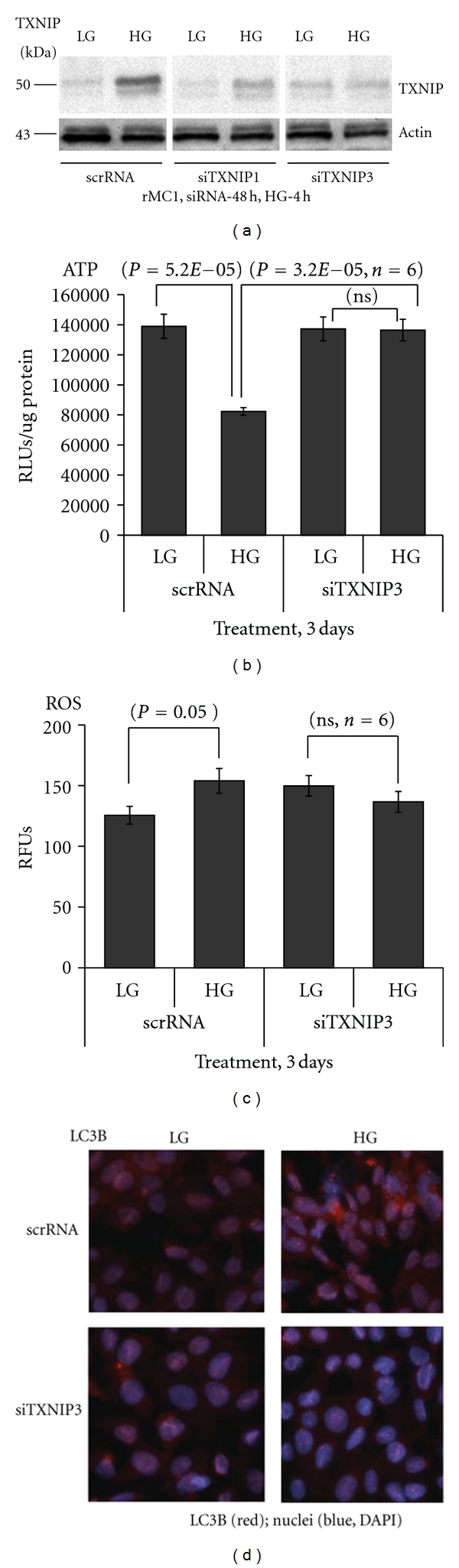
TXNIP knockdown by siRNA blocks HG-mediated ATP reduction, ROS generation, and LC3B expression in rMC1. (a) Seventy to eighty percent confluent rMC1 cells were transfected transiently with an scramble siRNA (scrRNA, control) or with TXNIP mRNA-targeted siRNAs-siTXNIP1 and siTXNIP3 (20 nM each) using HiPerfect transfection reagent in six-well plates or 60 mm culture plates in duplicates. After 6 h, complete media containing 5% serum were replaced and kept for 24 h. Media was subsequently changed to low serum media (0.2% serum) for 48 h. Afterwards, HG was added for 4 h and TXNIP amount was measured by Western blotting. We observe that siTXNIP3 gives a consistent suppression of TXNIP (~70%) and used in further studies. A representative blot of *n* = 4 is shown here. Actin was used as a control for protein loading and siRNA specificity. (b) rMC1 cells were transiently transfected in 24-well plates with siTXNIP3 for 48 h and then HG was added for 72 h in low serum media. Intracellular ATP concentration was measured and normalized to protein concentration (RLUs/*μ*g protein). HG reduces ATP level in scrRNA-treated rMC1 cells while siTXNIP3 transfection blunted HG effects. (c) Similar to ATP determination, we also measured ROS levels after 72 h of HG exposure in both scrRNA and siTXNIP3-transfected rMC1 with CM-H2DCFDA. HG increases ROS levels in scrRNA cells (*P* < 0.05, *n* = 6) versus LG but this effect was not observed in siTXNIP3 cells. (d) LC3B staining under HG exposure for 5 days is also reduced in siTXNIP3-transfected cells when compared with the scrRNA cells under similar duration of HG exposure (right panels). Under LG, LC3B staining is minimal in both scrRNA and siTXNIP3-treated rMC1 cells after 5 days. A representative of *n* = 3 is shown here.

Finally, we asked to what extent the sustained induction of TXNIP in rMC1 under HG is responsible for metabolic dysregulation in rMC1. We used a transient transfection method of siRNA to knock down TXNIP and assess its effect on ATP and ROS levels and LC3B expression in rMC1 under HG. First, we showed that HG-induced TXNIP protein level is reduced by two different TXNIP siRNAs (siTXNIP1 and siTXNIP3). As shown in Figure 9(a), siTXNIP3 gave a consistent suppression of TXNIP. Conversely, HG is still able to induce TXNIP expression in scrRNA-transfected cells. Therefore, we used the scrRNA (control) and siTXNIP3 in further studies. We observe that HG reduces ATP level significantly (*P* = 5.2*E* − 05, *n* = 6) in scrRNA-transfected cells, which is absent in siTXNIP3-transfected rMC1 cells (Figure 9(b)). In addition, HG-induced ROS generation in scrRNA-transfected cells is also blocked by siTXNIP3 (Figure 9(c)). Furthermore, the staining of autophagic marker LC3B is increased by HG in scrRNA-treated cells, which is reduced by siTXNIP3 (Figure 9(d)). The results demonstrate that TXNIP mediates, at least in part, cellular oxidative stress, ATP reduction, and an autophagic response in retinal Muller glia under chronic hyperglycemia.

## 4. Discussion

Muller glia plays an important function in retinal tissue homeostasis by taking up glucose and nutrients from the blood. It supplies metabolic products to neurons for proper functioning and participates in uptake and detoxification of neurotransmitters [5, 42]. Therefore, an injury to Muller glia itself and sustained gliosis will produce proinflammatory cytokines and proangiogenic factors that may further affect the retinal vasculature and neurons leading to ocular complications of diabetes [43, 44]. TXNIP plays a critical role in retinal inflammation and apoptosis [9, 26, 45]. Here, we provide evidences that HG activates TXNIP in retinal Muller cells* in vitro* as well as *in vivo* in the diabetic rat retina (this study, [9]). We demonstrate a temporal response of Muller glia to chronic HG exposure and programming of a series of innate host defense mechanisms which involve (i) sustained TXNIP up-regulation, (ii) innate immune/ER stress responses—(iii) ATP reduction, (iv) oxidative/nitrosative stress, (v) hypoxic-like response, (vi) autophagy/mitophagy, (vii) inflammation and, (viii) an apoptotic signal. Thus, a temporal and spatial survival response of Muller cells to chronic hyperglycemia emerges and TXNIP may orchestrate some of these events. Definitely, these observations will have important implications toward understanding the molecular basis for disease development and progression of DR and in designing stage-specific therapeutic strategies to reduce retinal inflammation and neurovascular dysfunction in diabetes. 

TXNIP is considered as a pro-oxidative stress, pro-inflammatory, and proapoptotic protein under chronic hyperglycemia, diabetes, and cellular stress [9, 11, 13, 26, 46]. However, these deleterious effects of HG in cells are observed at later stages of sustained hyperglycemic exposure while TXNIP is an early response gene to HG and tissue injury. Therefore, TXNIP may be considered as an early sensor of metabolic and cellular danger and a significant component of the innate host defense mechanism(s) and wound healing processes [10, 15, 16]. In support of this hypothesis, we recently showed that TXNIP is responsible for S100B-RAGE axis-induced expression of the innate immune response mediator, pro-IL-1*β*, and secretion of mature IL-1*β* in primary Schwann cells in culture and partial sciatic nerve injury recovery [10]. Under these conditions, an increase in ROS generation was not observed [8, 10]. This finding is in agreement with our current data showing that HG induces TXNIP and pro-IL-1*β* expression and activation of NLRP3 inflammasome and caspase-1 at 4–24 h without ROS generation (Figures 2 and 3). In fact, this is also similar to the condition of chronic Granulomatous Disease where impaired ROS production due to a genetic defect does not affect IL-1*β* secretion [47, 48].

 In acute infection models using circulating monocytes and stromal macrophages, however the NLRP3 inflammasome assembly, caspase-1 activation, and pro-IL-1*β* processing and secretion require ROS generation and ATP release [15, 49–51]. According to this model, during oxidative stress, ROS dissociates TXNIP bound to TRX. TXNIP then binds to NLRP3 to mediate NLRP3 inflammasome assembly containing ASC (Apoptotic Speck Protein Containing a Caspase Recruitment Domain (CARD)) and procaspase-1 [15, 16]. This subsequently leads to caspase-1 autocleavage and activation. Activated caspase-1 then processes pro-IL-1*β* to its mature form and is then secreted extracellularly. IL-1*β* is a potent pro-inflammatory cytokine and, upon release, induces several pro-inflammatory cytokines and chemokines. Therefore, IL-1*β* expression is highly regulated at multiple steps including at least two signals—a priming and a processing/maturation—while the mechanism of extracellular secretion is yet to be determined [52]. 

In our study, pro-IL-1*β*, NLRP3 inflammasome and procaspase-1 levels oscillate at 4 h and day 3 of HG exposure. The first response at 4 h occurs in the absence of ROS release as discussed previously while the second response at day 3 occurs under ROS/oxidative stress. We and others [10, 21] showed that TXNIP is responsible for pro-IL-1*β* priming/expression but the role of TXNIP in NLRP3 inflammasome assembly and caspase-1 activation is yet to be resolved as conflicting data exist at present [15, 21, 53]. Therefore, further studies will focus on the mechanism(s) of NLRP3 inflammasome activation and pro-IL-1*β* processing and the role of TXNIP in this process in the diabetic retina and under chronic hyperglycemia. 

Nutrient excess (glucose availability) leads to increase rates of glycolysis and energy (ATP) production, which is evident from our data showing elevated intracellular ATP levels at 4 h. Mitochondrial ATP synthesis produces ROS during electron transfer and OxPhos at ETC in the mitochondrial inner membrane. Furthermore, glucose and ATP induce increased protein synthesis in the ER lumen and ER stress leading to a UPR response to enhance protein folding and anti-oxidant capacity via an induction of mitochondrial and cytosolic superoxide dismutases and catalase [40]. Indeed, we observed an activation of the IRE1/XBP1 pathway in rMC1 by HG, which is demonstrated by the enhanced DNA-binding activity of nuclear sXBP1 in EMSA (Figure 8(c)). sXBP1 is known to activate the transcription of anti-oxidant genes including those stated above [40]. This may partly explain why we observed a decrease in ROS level at 4 and 24 h of HG treatment in spite of TXNIP up-regulation and IL-1*β* expression. Nonetheless, as hyperglycemia persists, the sXBP1 activity reduces and the defensive program is switched to a reduction in cellular ATP content, potentially in an attempt to minimize mitochondrial ROS generation through the aerobic respiration [54]. This may activate the cytosolic anaerobic glycolysis via an inhibition of pyruvate kinase at TCA cycle and lactate production, which is a survival response for phagocytic and cancer cells in a hypoxic environment during inflammation and tumor progression [55, 56]. Even then, the ETC in mitochondrial inner membrane continues to leak electrons and produces ROS at complexes I and III [57]. This eventually will lead to accumulation of ROS since TXNIP up-regulation can reduce the ROS scavenging capacity of TRX. Such an event seems to occur at day 3 of HG exposure in rMC1s, where there is a second surge in IL-1*β* and NLRP3 expression is observed (Figure 3(d)). The surge may be a second attempt to correct cellular metabolic defects and induce cell survival. However, these responses cause a further decline in cellular ATP level evoking a hypoxic-like response (HIF-1*α* activation) under hyperglycemia and normoxia (Figure 8(d)). 

Sustained TXNIP up-regulation may establish an oxidative stress environment in rMC1 at later stages of HG exposure (Figure 4(a)) after an initial innate immune response, ER stress, and ATP reduction. As ROS and iNOS expressions increase at day 3, nitrosative stress and protein thiol S-nitrosylation (SNO) as well as tyrosine nitration activities will be enhanced [58–60]. Initially, SNO modification of pro-apoptotic caspase-3 prevents its release from mitochondria [60]; however continued ROS/RNS will damage proteins, nucleic acids, and organelles including ER and mitochondria. Therefore, another ancient survival program of autophagy (mostly activated under nutrient starvation to recycle protein aggregates and organelles) and mitophagy (removal of damaged mitochondria by autophagic processes) is activated [36–39]. This is supported by our observation that in spite of the significant increase in ROS levels (day 3 to 5 of HG), activity of mitochondrial membrane enzyme cytochrome c oxidase at complex IV is sustained and cell viability is preserved. Preservation of cell viability is indicated by increased MTT that measures the reductase activity of succinate dehydrogenase at complex II. These findings suggest an active regeneration of mitochondria by removing the oxidatively damaged ones and provide cell viability. Such an assumption is supported by the observation that autophagic LC3B punctae are increased in rMC1 (Figure 6) and MTT activity is enhanced (Figure 5).

 The mechanism of autophagy initiation is complex and recent studies have pointed to a role of the nuclear DNA-binding protein HMGB1 under oxidative stress [60]. Nuclear TXNIP may cause HMGB1 oxidation (S-nitrosylation) and nuclear export [61, 62]), which participates in beclin 1 dissociation from antiapoptotic bcl2 and initiation of autophagy [38, 39]. Oxidation of HMGB1 at cysteine residues 23, 45, and 106, probably by inhibition of TRX by TXNIP in the nucleus, causes its translocation to the cytosol and interaction with beclin 1 [38, 39]. HMGB1 is an abundant nuclear protein considered to have a dual function: it acts (i) as a transcriptional activator in the nucleus by binding to linker DNA between nucleosomes inducing DNA bending and enhances transcription factor accessibility, and (ii) as a pro-inflammatory cytokine (passively or actively secreted) by injured and dying cells as a danger signal and damage-associated molecular pattern (DAMP) molecule, which binds to innate pattern recognition receptors (PRRs) such as TLR2, TLR4 and RAGE [20, 61]. (iii) In addition, HMGB1 may also have a role in autophagy in rMC1 (this study, [60]). Previously, we showed that RAGE activation by its endogenous ligand S100B activates TXNIP expression and induces pro-inflammatory genes for IL-1*β*, Cox-2, VEGF-A, ICAM1, and fibrotic FN [8–10]. While IL-1*β* is an early mediator of innate immunity and inflammation, HMGB1 is regarded as a late mediator in chronic inflammatory diseases [63, 64]. Thus, the HMGB1-TLR4 and/or HMGB1-RAGE axis may further activate TXNIP and IL-1*β* expression thereby potentiating a vicious cycle of innate immunity and sterile inflammation under chronic hyperglycemia. The fact that knockdown of TXNIP by siRNA blocks IL-1*β* expression in the retina *in vivo* (Figure 1(c)) and ATP depletion, ROS generation, and LC3B induction in rMC1 *in vitro* (Figure 9) suggest that TXNIP is critical for Muller glia activation, autophagy, and inflammation. TXNIP therefore may represent a potent gene and therapeutic target to prevent DR pathogenesis [8, 9]. 

Sustained ROS/RNS generation and activation of autophagy/mitophagy will eventually trigger an apoptotic signal and premature cell death due to excessive removal of essential organelles and proteins in the cell while damaged mitochondria leaks electrons [57, 58]. Mitochondrial oxidative/nitrosative stress causes ER stress, which in turn induces mitochondrial damage thus generating a vicious cycle of organelle damage and tissue injury [41]. Here, we show that increases in both TXNIP and proapoptotic caspase-3 occur at later periods of HG exposure for example, at day 5. Nonetheless, we propose that the ER stress and autophagic and apoptotic signal will be shifted towards a caspase-1/7-dependent pro-inflammatory cell death (pyroptosis) [65]. This is because apoptosis is an ATP-dependent (consuming) process and requires active ATP synthesis [65]. In the current study, we observe ATP depletion under sustained HG and a late surge in caspase-1 level at day 5. Furthermore, the caspase-3 probe, FLICA, used in this study may also recognize caspase-7, which is downstream of caspase-1 and closely related to caspase-3. In agreement with the idea of pyroptosis under HG, the dying and oxidatively stressed rMC1 produces pro-inflammatory genes such as TXNIP, iNOS, Cox-2, and proangiogenic VEGF-A (Figures 7(c)–7(f); [66]). The induction of VEGF-A is particularly interesting because of its association with ATP depletion and HIF-1*α* activation. VEGF-A is a potent vascular permeability and proangiogenic factor that is regulated by HIF-1*α* and implicated in diabetic vasculopathy [56, 67]. Protein S-nitrosylation also consumes molecular oxygen [58]; therefore, it may result in reduced mitochondrial ATP production and generation of an intracellular hypoxic-like environment inducing HIF-1*α* activation under normoxia. An understanding of the TXNIP-HIF-1*α*-VEGF-A axis and its temporal response under chronic hyperglycemia in Muller cells that enhances VEGF-A expression in diabetes will be important. It is generally accepted that Muller glia is the main source of VEGF-A in the neural retina [68, 69]. 

An accumulation of HIF-1*α* in diabetic retinas of STZ-mice was recently reported [67], which is also associated with an increase in VEGF-A expression. Furthermore, a disruption of the Hif-1*α* gene in Müller cells alleviates retinal inflammation and vascular leakage in diabetic Hif-1*α* KO mice, further indicating an important role of Müller glia-derived HIF-1*α*-VEGF-A axis in DR [67]. In addition, attenuation of ER stress by chemical chaperones such as 4-phenyl butaric acid reduces VEGF-A expression in retinal endothelial cells in culture induced by hypoxia as well as in a mouse model of oxygen-induced retinopathy (OIR) [23, 70, 71]. Thus, several reports have pointed to an important role of ER stress/UPR signaling in retinal inflammation, hypoxia, and perhaps autophagy. Nonetheless, a critical function of TXNIP in ER stress, autophagy, and apoptosis/pyroptosis in animal models of DR remains to be investigated, which will form the basis for a separate manuscript. Sustained and excessive induction of each of these cellular defensive/survival processes, including ER stress and UPR, will evoke chronic inflammation and DR pathogenesis. ER-stress, autophagy, and innate immune responses are survival mechanisms; however prolonged induction of these cellular processes will ultimately lead to premature cell death and disease progression of microvascular complications of diabetes. Cellular DAMPs (ATP, uric acid, HMGB1, mitochondrial DNA, etc.) released by injured and dying cells are recognized by innate PRRs such as TLR4, TLR9, RAGE, P2X7R, and NLRP3 inflammasome and together the DAMPs and PRRs may play causative roles in retinal ER-stress, autophagy, inflammation, and DR pathogenesis. 

**Figure 10:**
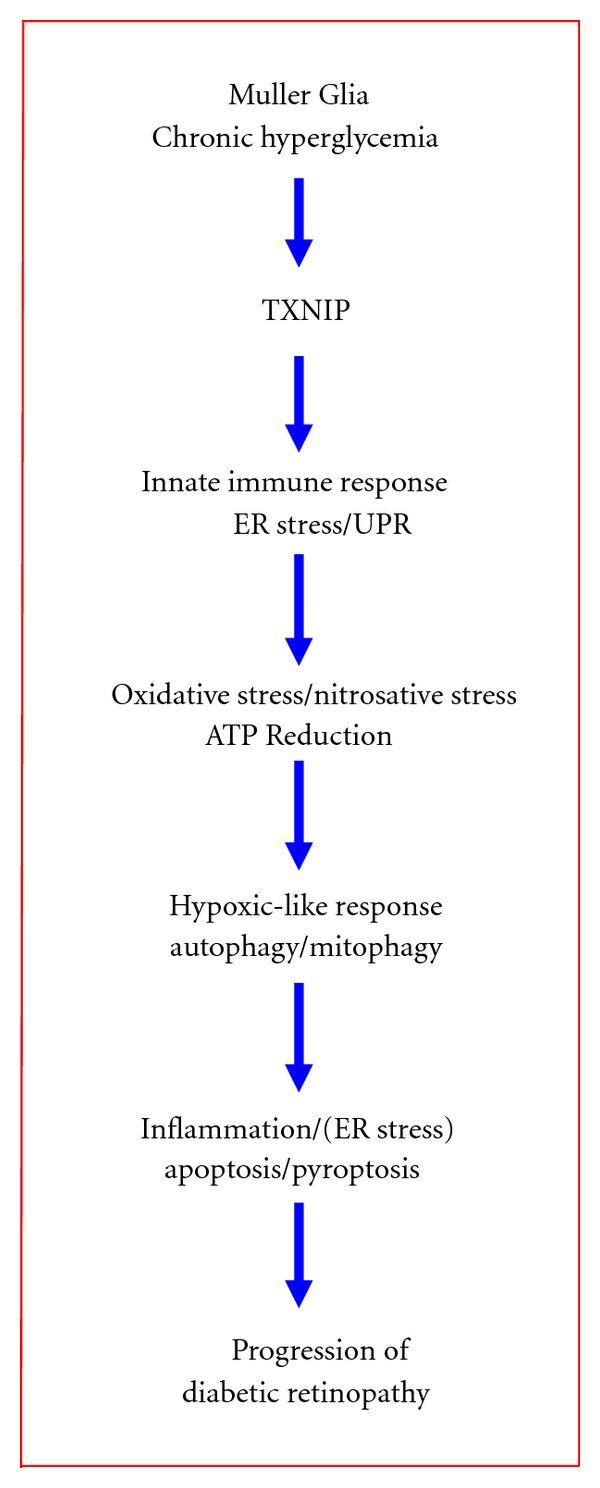
A schematic summary of potential cellular responses by retinal Muller glia in chronic hyperglycemia and diabetes. The sequence of molecular events that retinal Muller cells react to chronic hyperglycemia include (i) sustained upregulation of TXNIP, (ii) an initial innate immune and UPR response to excess glucose metabolism and oxidative phosphorylation (ATP generation), (iii) oxidative stress (ROS/RNS generation) and a hypoxia-like response through ATP reduction, (iv) an induction of an autophagic-mitophagic pathway, and (v) ER-stress and inflammation. These cellular responses constitute intrinsic cell survival/defense mechanisms, which, under chronic cell stress and injury, may promote premature cell death and disease progression of DR.

In conclusion, we show for the first time that HG sustains TXNIP expression in rat Muller glia and orchestrates a duration-dependent cellular program of innate host defense and survival mechanisms that culminate in oxidative stress, ER stress, autophagy, inflammation, and cell death. While detailed mechanisms are yet to be worked out, a temporal pattern emerges here as to how retinal Muller glia might respond to chronic hyperglycemia in diabetes. These include (i) sustained TXNIP up-regulation, (ii) an initial innate immune and ER stress response to excess glucose metabolism (ATP generation), (iii) oxidative stress and a hypoxia-like response through ATP reduction, (iv) induction of an autophagic-apoptosis pathway, and (v) inflammation. A temporal response of Muller glia to chronic hyperglycemia and potential molecular events are summarized in Figure 10. These findings clearly point to a crucial role of TXNIP in Muller glia activation, oxidative/nitrosative, and ER stress, and sterile inflammation under chronic hyperglycemia and suggest a potential gene and drug target for preventing neurovascular injury/cell death and pathogenesis of DR. Lastly, while our manuscript is in submission, an article came out to demonstrate purinergic and glycemic induction of TXNIP in rMC1 in culture [72], which also supports our findings in the present study.
